# Supplementary material for: Radical-constructed intergrown titanosilicalite interfaces for efficient direct propene epoxidation with H2 and O2
Source: Nat Commun. 2025 Jul 1;16:5515. doi: 10.1038/s41467-025-60637-0 (PMC12215953; doi:10.1038/s41467-025-60637-0)
Supplement: Supplementary file 1 — Supplementary Information [file 41467_2025_60637_MOESM1_ESM.pdf]

Supplementary Information

Radical-constructed Intergrown Titanosilicalite Interfaces for Efficient Direct Propene Epoxidation with H_2 and O_2

Dong Lin[1,2], Xiang Feng[1,*], Yang Xu[1], Richard J. Lewis[2,*], Xiao Chen[3], Thomas E. Davies[2], Samuel Pattisson[2], Mark Douthwaite[2], Defu Yin[1], Qiuming He[1], Xiuhui Zheng[1], De Chen[4], Chaohe Yang[1] and Graham J. Hutchings[2,*]

[1]*State Key Laboratory of Heavy Oil Processing, China University of Petroleum, Qingdao 266580, China*

[2]*Max Planck- Cardiff Centre on the Fundamentals of Heterogeneous Catalysis FUNCAT, Cardiff Catalysis Institute, School of Chemistry, Cardiff University, Cardiff, CF24 4HQ, United Kingdom*

[3]*Beijing Key Laboratory of Green Chemical Reaction Engineering and Technology, Department of Chemical Engineering, Tsinghua University, Beijing 100084, China*

[4]*Department of Chemical Engineering, Norwegian University of Science and Technology, Trondheim 7491, Norway*

Corresponding author

Correspondence to: Xiang Feng (xiangfeng@upc.edu.cn); Richard J. Lewis (lewisr27@cardiff.ac.uk); Graham J. Hutchings (hutch@cardiff.ac.uk)

Table of Contents

1. Reagents S3

2. Supplementary Tables S4

Total acid density of different TS-1 samples S4

The relative content of Si/Ti, bulk Si/Ti ratios and Au contents in different framework topologies S5

The pore properties of different titanosilicalites before and after Au loading S5

A comparison of catalytic performance towards the in situ epoxidation of propene S6

3. Supplementary Figures S8

In-situ UV-vis-NIR system S8

XRD patterns of different TS-1 samples S9

The FT-IR spectra of different TS-1 samples S10

NH_3-TPD profiles of different TS-1 samples S11

UV-vis spectra of TS-1 different samples S12

UV-Raman spectra of TS-1 different samples S13

Py-IR spectra of different TS-1 samples S14

Typical SEM images of different zeolites S15

The reaction performance of Au/TS-1-1000W S16

Typical HRTEM images of different catalysts S17

The Au 4*f* spectra of different catalysts S18

The TGA profiles of different catalysts S19

The C1*s* XPS spectra of different catalysts S20

The reaction performance of different catalysts S21

Typical STEM images and associated particle count histograms of different catalysts S22

The productivity of H_2O_2 on different Au/TS-1 catalysts S23

Free energies of adsorption on TiO_4 and TiO_3 sites S24

In-situ UV-vis spectra of different catalysts S25

The hydrogen efficiency of different catalysts S26

The electronic structure of different Ti sites S27

1. Reagents

Propene (C_3H_6, 99.999 %, Shanghai Shenkai Gases Technology Co., Ltd)

Hydrogen (H_2, 99.999 %, Shanghai Shenkai Gases Technology Co., Ltd)

Oxygen (O_2, 99.999 %, Shanghai Shenkai Gases Technology Co., Ltd)

Nitrogen (N_2, 99.999 %, Shanghai Shenkai Gases Technology Co., Ltd)

Tetrapropylammonium hydroxide solution (TPAOH, 25 wt%, Sinopharm Chemical Reagent Co., Ltd)

Tetraethyl orthosilicate (TEOS, 98.5 wt%, Sinopharm Chemical Reagent Co., Ltd)

Tetrabutyl titanate (TBOT, 99.0 wt%, Shanghai Aladdin Biochemical Technology Co., Ltd)

Chloroauric acid ($HAuCl_4 \cdot 4H_2O$, 99.9 wt %, Sinopharm Chemical Reagent Co., Ltd)

Sodium carbonate (Na_2CO_3, 99.5 wt%, Shanghai Aladdin Biochemical Technology Co., Ltd)

2. Supplementary Tables

Table S1 Total acid density of different TS-1 samples.

Samples	Acid density (mmol/g)
TS-1-200W	2.67×10^{-2}
TS-1-300W	2.06×10^{-2}
TS-1-500W	1.84×10^{-2}
TS-1-1000W	1.58×10^{-2}

Table S2 The relative content of Si/Ti, bulk Si/Ti ratios and Au contents in different framework topologies.

	SiO_2 (wt%)	TiO_2 (wt%)	Si/Ti molar ratio[a]	Au loading (wt%)[b]
TS-1-200W	99.30	0.62	211	0.07
TS-1-300W	98.84	0.63	207	0.07
TS-1-500W	99.24	0.68	192	0.07
TS-1-1000W	99.32	0.66	201	0.07

[a]The bulk Si/Ti ratios were determined by the XRF results.

[b]The bulk Au loadings were determined by the ICP results.

Table S3 The pore properties of different titanosilicalites before and after Au loading.

Samples	$V_{micro}{}^{a}$ (cm^3g^{-1}) before Au loading	$V_{micro}{}^{a}$ (cm^3g^{-1}) after Au loading	$V_{na}{}^{b}$	$V_{na}/V_{support}{}^{c}$
TS-1-200W	0.194	0.189	0.005	0.974
TS-1-300W	0.191	0.188	0.003	0.984
TS-1-500W	0.189	0.181	0.008	0.958
TS-1-1000W	0.205	0.197	0.008	0.961

[a]V_{micro} represent the microporous volume before and after Au loading.

[b]V_{na} represent the volume not accessible to N_2 after Au loading. V_{na} is the difference between the micropore volumes of TS-1 support and Au/TS-1 catalyst.

[c]$V_{na}/V_{support}$ represent the effect of spatial location of gold for support volume.

The descriptor ($V_{na}/V_{support}$) serves as a tool for estimating the extent to which zeolite channels are occupied by the Au nanoparticles. Here, V_{na} denote the volume inaccessible to probe molecules (N_2). The volume not accessible to N_2 is calculated as the difference between the micropore volumes of the TS-1 support ($V_{support}$) and the Au/TS-1 catalyst.

Table S4 A comparison of catalytic performance towards the in situ epoxidation of propene.

Catalyst	Reaction conditions	PO formation rate ($g_{PO}h^{-1}g_{Au}^{-1}$)	PO formation rate ($g_{PO}h^{-1}kg_{Cat}^{-1}$)	Reference
Au/TS-1-500W	200°C, 0.15 g of catalyst, $H_2/O_2/C_3H_6/N_2$=1:1:1:7, space velocity: 14000 $mL{\cdot}h^{-1}{\cdot}g_{Cat}^{-1}$	455.0	318.5	This work
Au/TS-1-B	200°C, 0.15 g of catalyst, $H_2/O_2/C_3H_6/N_2$=1:1:1:7, space velocity: 14000 $mL{\cdot}h^{-1}{\cdot}g_{Cat}^{-1}$	151.5	151.5	[9]
Au/TS-2-B	200°C, 0.15 g of catalyst, $H_2/O_2/C_3H_6/N_2$=1:1:1:7, space velocity: 19200 $mL{\cdot}h^{-1}{\cdot}g_{Cat}^{-1}$	118	131	[46]
0.122Au/TS-1(99)	200°C, 0.15 g of catalyst, $H_2/O_2/C_3H_6/N_2$=1:1:1:7, space velocity: 14000 $mL{\cdot}h^{-1}{\cdot}g_{Cat}^{-1}$	69	84	[51]
0.143Au/TS-1(99)	200°C, 0.15 g of catalyst, $H_2/O_2/C_3H_6/N_2$=1:1:1:7, space velocity: 14000 $mL{\cdot}h^{-1}{\cdot}g_{Cat}^{-1}$	103	148	[51]
0.146Au/TS-1(99)	200°C, 0.15 g of catalyst, $H_2/O_2/C_3H_6/N_2$=1:1:1:7, space velocity: 14000 $mL{\cdot}h^{-1}{\cdot}g_{Cat}^{-1}$	105	154	[51]
Au/HTS-1(NIMG)	200°C, 0.15 g of catalyst, $H_2/O_2/C_3H_6/N_2$=1:1:1:7, space velocity: 14000 $mL{\cdot}h^{-1}{\cdot}g_{Cat}^{-1}$	150.3	150.3	[52]
Au–Ti@MFI	300°C, 0.15 g of catalyst, $H_2/O_2/C_3H_6/N_2$=1:1:1:7, space velocity: 7000 $mL{\cdot}h^{-1}{\cdot}g_{Cat}^{-1}$	41	205	[53]
Au/TS-1-B	200°C, 0.15 g of catalyst, $H_2/O_2/C_3H_6/N_2$=1:1:1:7, space velocity: 14000 $mL{\cdot}h^{-1}{\cdot}g_{Cat}^{-1}$	267	240	[54]
Au/TS-1-B(4)	200°C, 0.15 g of catalyst, $H_2/O_2/C_3H_6/N_2$=1:1:1:7, space velocity: 14000 $mL{\cdot}h^{-1}{\cdot}g_{Cat}^{-1}$	160	144	[55]

0.05Au/U-TS-1(119)	200ºC, 0.15 g of catalyst, $H_2/O_2/C_3H_6/N_2$=1:1:1:7, space velocity: 14000 $mL{\cdot}h^{-1}{\cdot}g_{Cat}^{-1}$	130	65	[56]
0.07Au/U-TS-1(119)	200ºC, 0.15 g of catalyst, $H_2/O_2/C_3H_6/N_2$=1:1:1:7, space velocity: 14000 $mL{\cdot}h^{-1}{\cdot}g_{Cat}^{-1}$	127.1	89	[56]
0.09Au/U-TS-1(119)	200ºC, 0.15 g of catalyst, $H_2/O_2/C_3H_6/N_2$=1:1:1:7, space velocity: 14000 $mL{\cdot}h^{-1}{\cdot}g_{Cat}^{-1}$	106.7	96	[56]
0.14Au/TS-1(121)Cs	200ºC, 0.15 g of catalyst, $H_2/O_2/C_3H_6/N_2$=1:1:1:7, space velocity: 14000 $mL{\cdot}h^{-1}{\cdot}g_{Cat}^{-1}$	240.7	337	[57]
0.76Au/TS-1(77)Cs	200ºC, 0.15 g of catalyst, $H_2/O_2/C_3H_6/N_2$=1:1:1:7, space velocity: 14000 $mL{\cdot}h^{-1}{\cdot}g_{Cat}^{-1}$	41.3	314	[57]
0.04Au/TS-1(121)Cs	200ºC, 0.15 g of catalyst, $H_2/O_2/C_3H_6/N_2$=1:1:1:7, space velocity: 14000 $mL{\cdot}h^{-1}{\cdot}g_{Cat}^{-1}$	245	98	[57]
Au/TS-1-B (S-Na)	200ºC, 0.15 g of catalyst, $H_2/O_2/C_3H_6/N_2$=1:1:1:7, space velocity: 14000 $mL{\cdot}h^{-1}{\cdot}g_{Cat}^{-1}$	297	89.1	[58]
0.23Na-Au/TS-1	200ºC, 0.15 g of catalyst, $H_2/O_2/C_3H_6/N_2$=1:1:1:7, space velocity: 14000 $mL{\cdot}h^{-1}{\cdot}g_{Cat}^{-1}$	310	210	[58]

3. Supplementary Figures

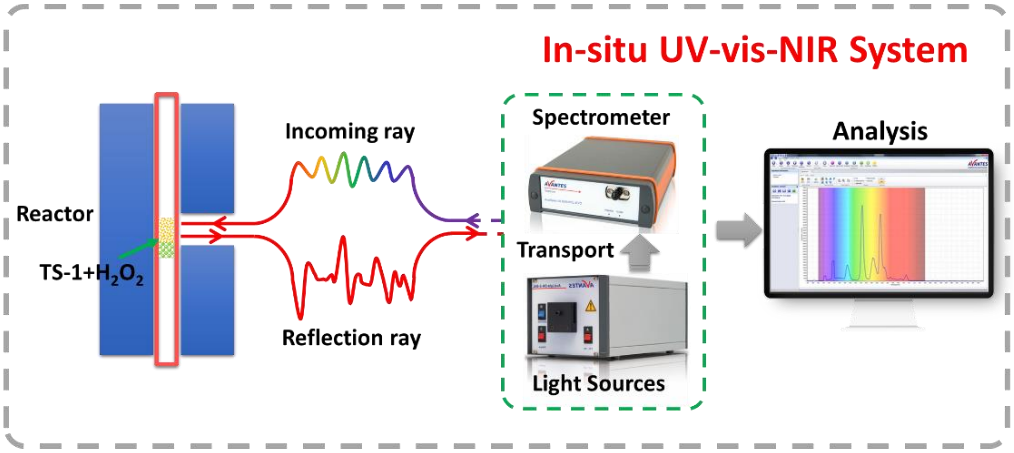

Figure S1 In-situ UV-vis-NIR system. The schematic diagram of the in-situ UV-vis-NIR system.

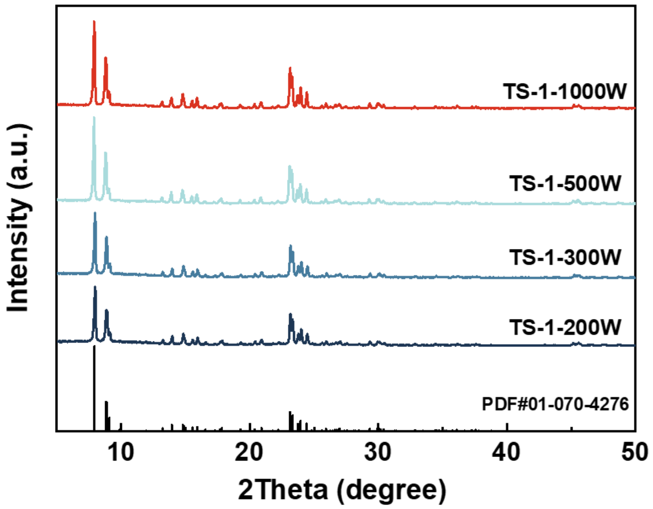

Figure S2 XRD patterns of different TS-1 samples. The XRD diffractograms of TS-1-200 W, TS-1-300 W, TS-1-500 W and TS-1-1000 W.

All TS-1 samples exhibit identical diffraction peaks, situated at 2θ of 7.9, 8.8, 23.1, 23.9, and 24.3°, thereby confirming the presence of the MFI topological structure. Moreover, in all TS-1 samples, a single peak is observed at 2θ = 24.3°, affirming the presence of an orthorhombic unit cell in the structure. In the XRD spectra, it is evident that there are no significant peaks at 25.4° , suggesting that all samples either contain minimal or no TiO_2 species.

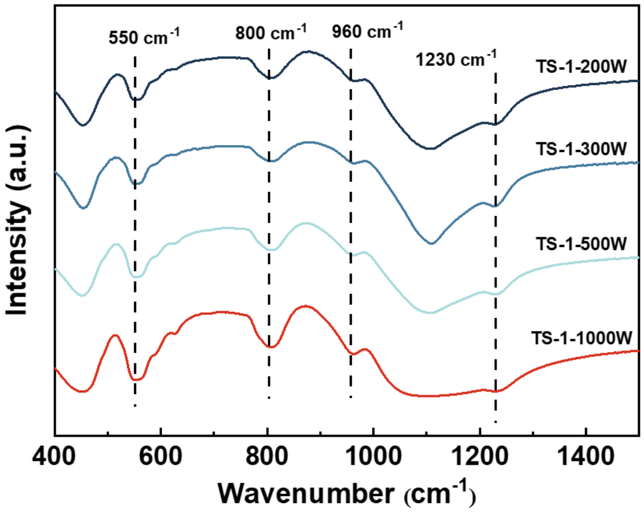

Figure S3 The FT-IR spectra of different TS-1 samples. The FT-IR spectra of The FT-IR spectra of TS-1-200W, TS-1-300W, TS-1-500W and TS-1-1000W.

The absorption band observed at 550 cm^{-1} can be attributed to the vibration of the double five-membered ring unit, providing further evidence for the presence of the MFI structure, which aligns with the XRD findings. The bands appearing at 800 cm^{-1} correspond to the antisymmetrical stretching vibrations of the [SiO_4] units. Additionally, the band observed at 1230 cm^{-1} is associated with the asymmetrical stretching vibration of the MFI framework structure. The presence of the 960 cm^{-1} band is often considered as indicative of Ti substitution within the framework, occurring in the form of Ti-O-Si.

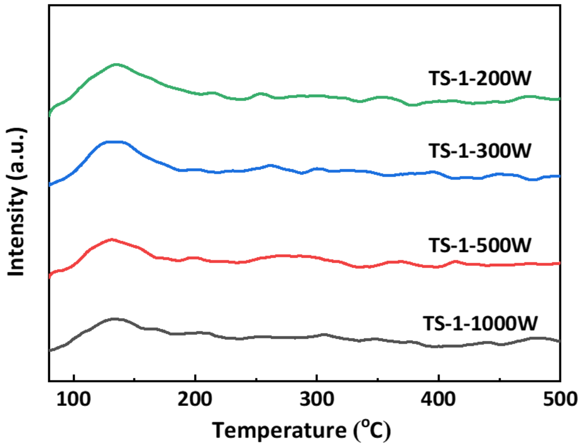

Figure S4 NH_3-TPD profiles of different TS-1 samples. NH_3-TPD profiles of different TS-1 samples synthesized with various power levels (ranging from 200 to 1000 W).

The NH_3-TPD analysis was employed to assess the presence of acid sites in all TS-1 samples. As depicted in Figure S4, only one NH_3 desorption peak at 135 °C is evident across all TS-1 samples, indicating the existence of a minimal quantity of weak acid centers within TS-1 materials.

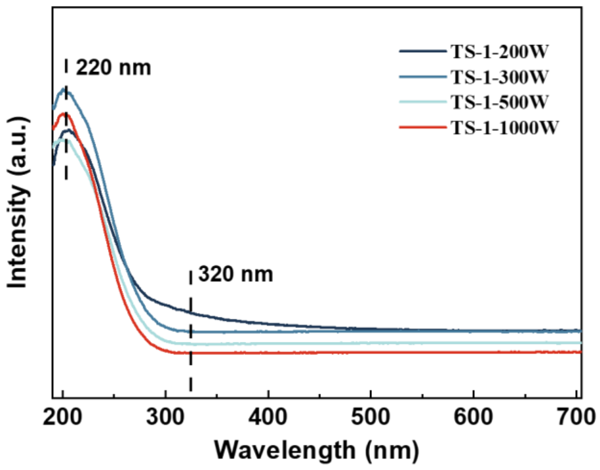

Figure S5 UV-vis spectra of different TS-1 samples. UV-vis spectra of TS-1-200W, TS-1-300W, TS-1-500W, TS-1-1000W.

The presence of Ti within the TS-1 framework is critical for its catalytic performance, a feature discernible through UV-vis characterization. The observation of a single adsorption peak at approximately 220 nm for all samples distinctly confirms the presence of isolated Ti(IV) species within the MFI framework. Notably, adsorption peaks associated with anatase TiO_2 species were detected at 330 nm in TS-1-200W. No adsorption peaks associated with anatase TiO_2 species were detected at 330 nm in TS-1-300W, TS-1-500W and TS-1-1000W.

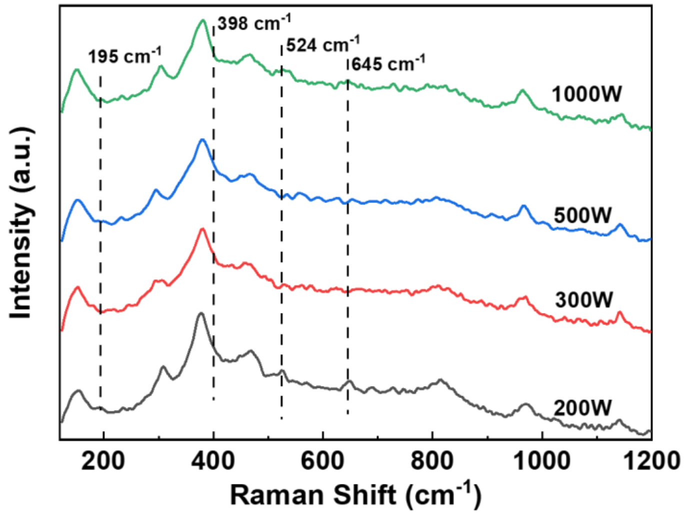

Figure S6 UV-Raman spectra of different TS-1 samples. UV-Raman spectra of TS-1-200W, TS-1-300W, TS-1-500W, TS-1-1000W.

It is noteworthy that only TS-1-200W exhibits small peaks at 195, 398, 524 and 645 cm^{-1}, suggesting the presence of anatase TiO_2. These observations align with the results from UV-vis spectroscopy, XPS, and SEM, providing additional evidence that TS-1-200W contains TiO_2 species, whereas other samples do not.

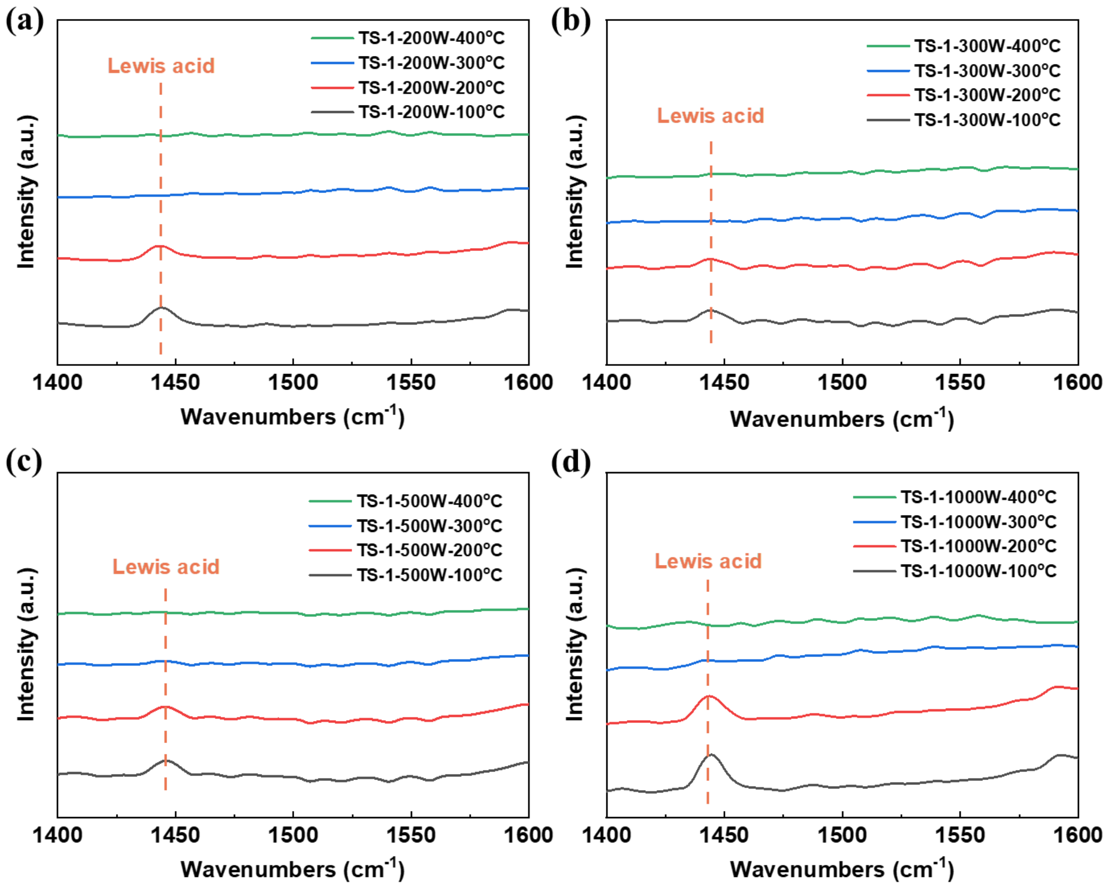

Figure S7 Py-IR spectra of different TS-1 samples. Py-IR spectra of different TS-1 samples synthesized with various power levels: (a) TS-1-200W, (b) TS-1-300W, (c) TS-1-500W and (d) TS-1-1000W.

The peaks at 1447 and 1601 cm^{-1} are ascribed to Lewis acid sites (LA). Additionally, the peaks at 1550 and 1490 cm^{-1} were attributed to Brønsted acid sites (BA) and the combination of Brønsted and Lewis acid sites (BA+LA), respectively. It is noteworthy that all titanosilicalites display prominent peaks of Lewis acid sites, linked to the framework Ti sites.

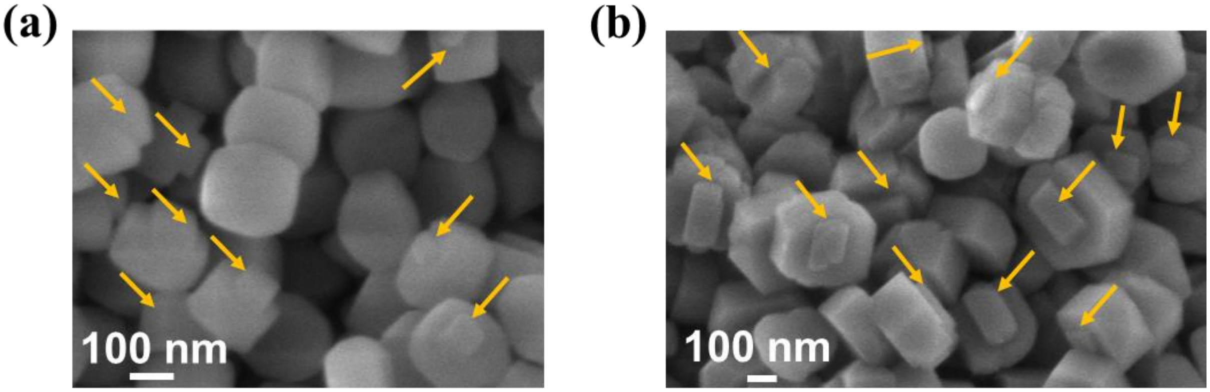

Figure S8 Typical SEM images of different zeolites. Typical SEM images of (a) pure siliceous S-1 and (b) Al-containing ZSM-5 samples.

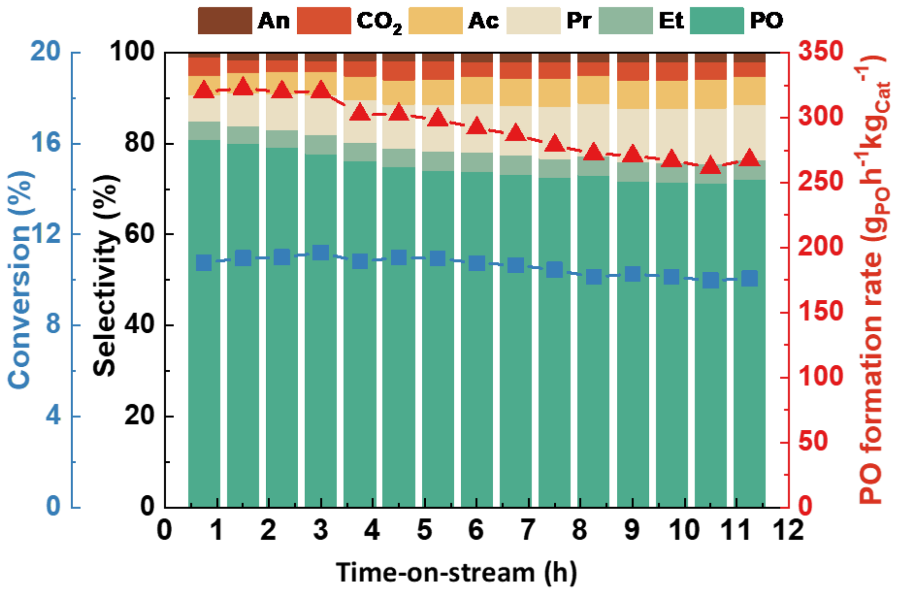

Figure S9 The reaction performance of Au/TS-1-1000W. The PO formation rate, propene conversion and reaction selectivity of Au/TS-1-1000W. Direct propene epoxidation with H_2 and O_2 conditions: reaction temperature (200°C), catalyst (0.15 g), $H_2/O_2/C_3H_6/N_2$=1:1:1:7, space velocity of 14000 $mL{\cdot}h^{-1}{\cdot}g_{Cat}^{-1}$.

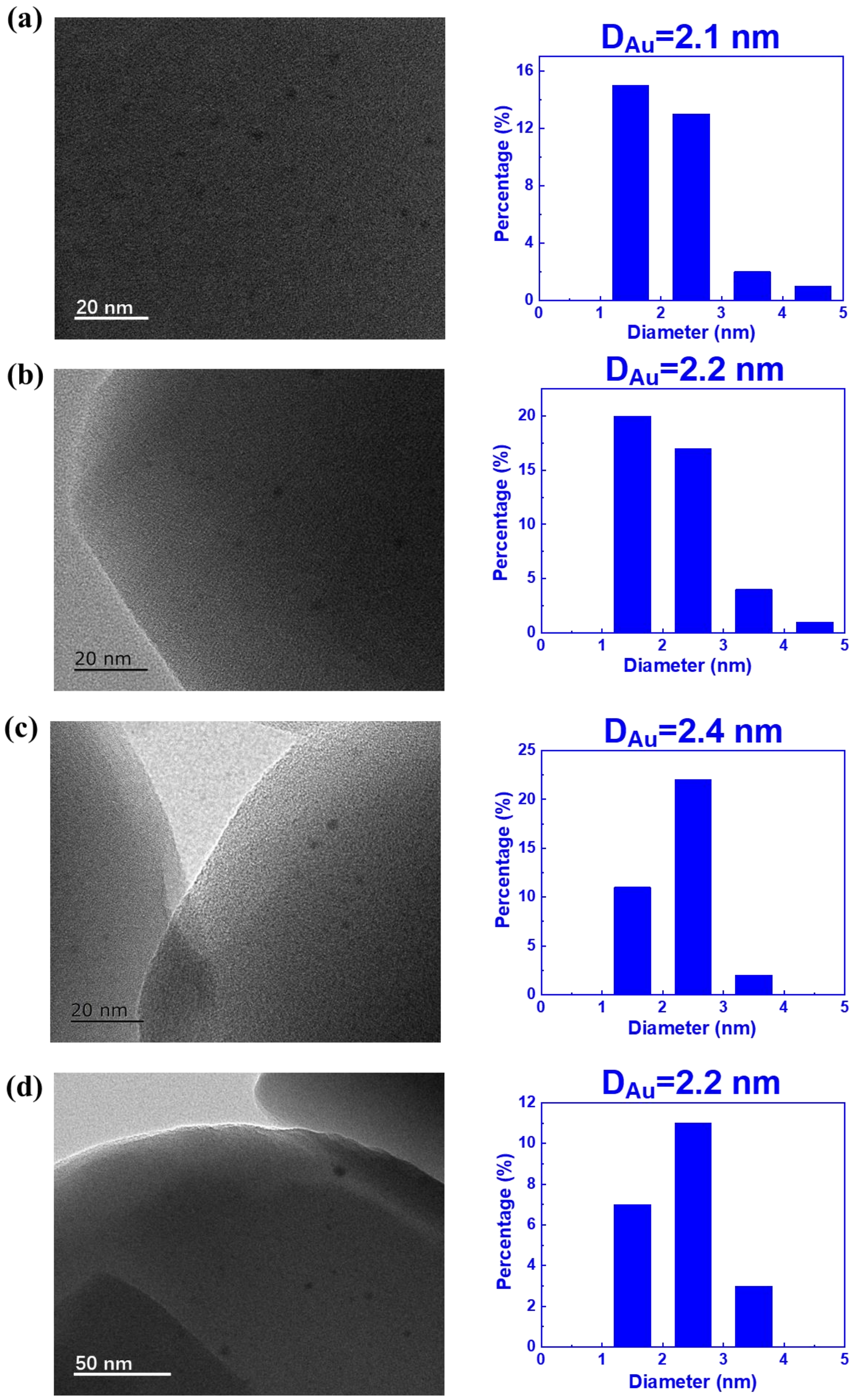

Figure S10 Typical HRTEM images of different catalysts. Typical HRTEM images of (a) Au/TS-1-200W, (b) Au/TS-1-300W, (c) Au/TS-1-500W and (d) Au/TS-1-1000W. The insets show the Au size distributions of all catalysts.

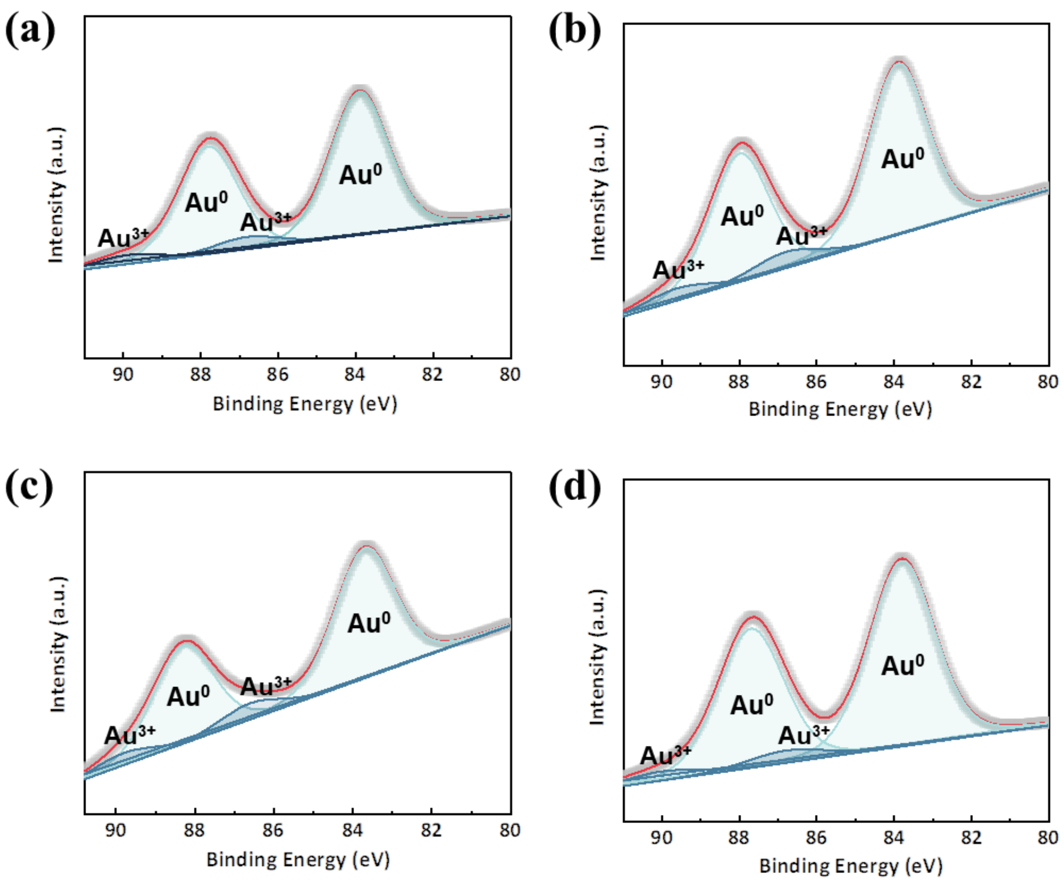

Figure S11 The Au 4*f* spectra of different catalysts. The Au 4*f* spectra of (a) Au/TS-1-200W, (b) Au/TS-1-300W, (c) Au/TS-1-500W and (d) Au/TS-1-1000W.

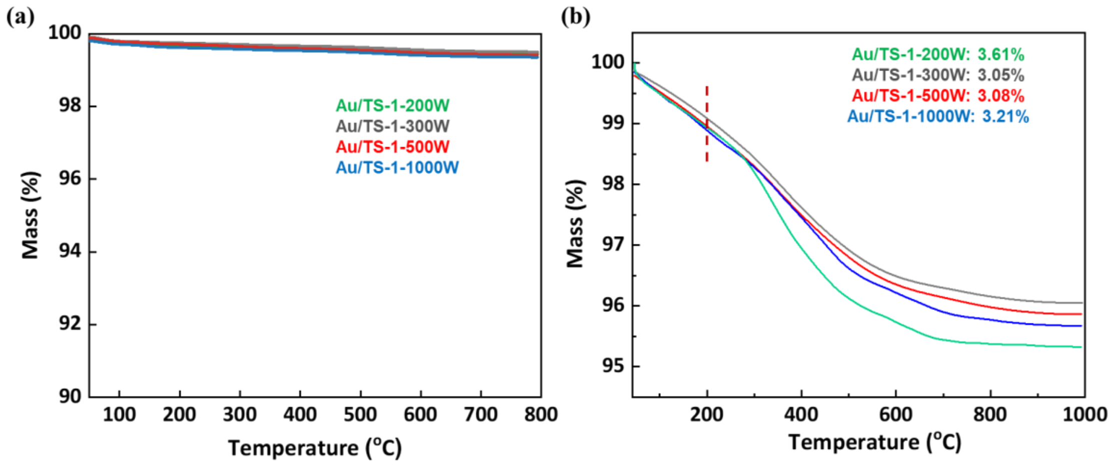

Figure S12 The TGA profiles of different catalysts. The TGA profiles of different (a) fresh catalysts and (b) catalysts after propene epoxidation at 200 °C.

The thermogravimetry analysis combined (TG, Netzsch) was used to reveal the coke content and coke species of the catalyst with a heating speed of 10°C/min from 30 to 1000°C. The weight loss occurring between 200-800°C and below 200°C is attributed to carbonaceous deposits and water, respectively.

The relative content of Si-OH (Q_3/Q_4) decreased from 22.1% to 17.0% (Figure 1f-1i) with the increase of power levels (ranging from 200 to 1000 W). TS-1-200W exhibited the highest Q_3/Q_4 ratio (22.1%), and a higher Q_3/Q_4 ratio indicates lower hydrophobicity, which is considered to hinder the desorption of propylene oxide (PO) molecules and promote the formation of carbonaceous species. As a result, Au/TS-1-200W demonstrated the highest weight loss (~3.61%) after propene epoxidation with H_2 and O_2.

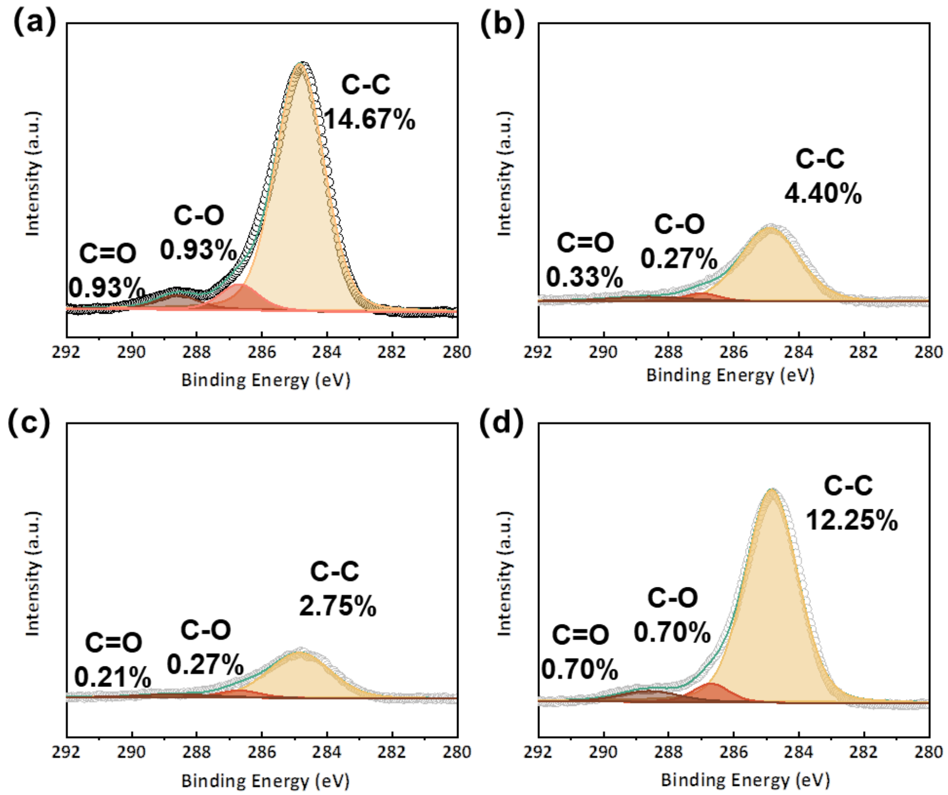

Figure S13 The C1*s* XPS spectra of different catalysts. The C1*s* XPS spectra of (a) Au/TS-1-200W, (b) Au/TS-1-300W, (c) Au/TS-1-500W and (d) Au/TS-1-1000W.

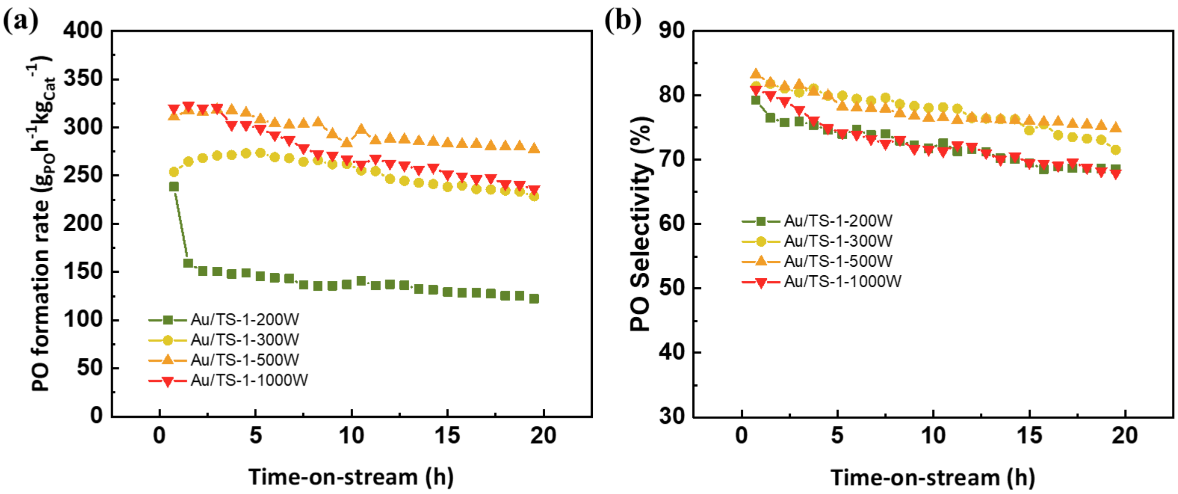

Figure S14 The reaction performance of different catalysts. The (a) PO formation rate and (b) reaction selectivity of different Au/TS-1 catalysts. Direct propene epoxidation with H_2 and O_2 conditions: reaction temperature (200°C), catalyst (0.15 g), $H_2/O_2/C_3H_6/N_2$=1:1:1:7, space velocity of 14000 mL·h^{-1}·g$_{Cat}$$^{-1}$.

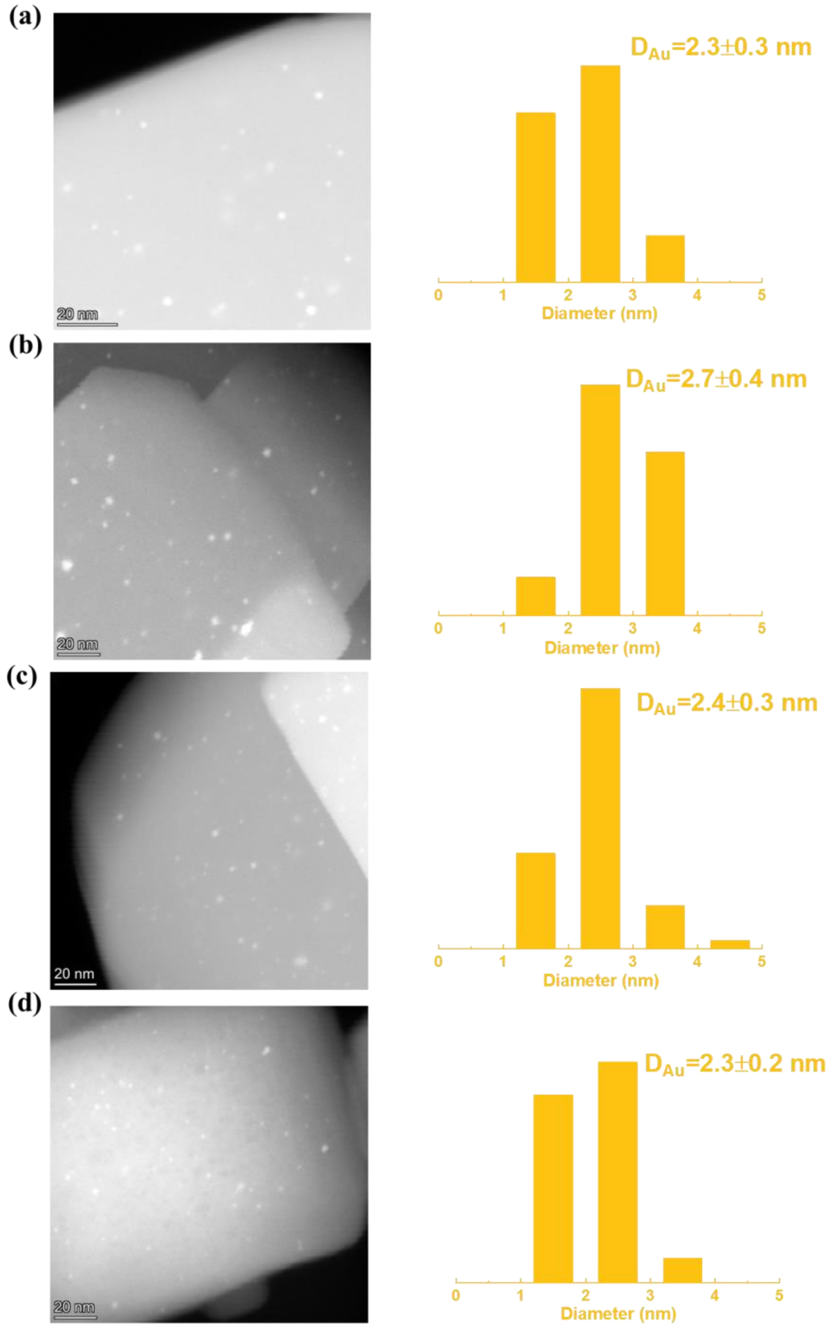

Figure S15 Typical STEM images and associated particle count histograms of different catalysts. Typical STEM images and associated particle count histograms of **(a)** Au/TS-1-200W, **(b)** Au/TS-1-300W, **(c)** Au/TS-1-500W and **(d)** Au/TS-1-1000W after reaction. Direct propene epoxidation with H_2 and O_2 conditions: reaction temperature (200°C), catalyst (0.15 g), $H_2/O_2/C_3H_6/N_2$=1:1:1:7, space velocity of 14000 mL·h^{-1}·g$_{Cat}^{-1}$.

Post-reaction HRTEM analysis of the different Au/TS-1 catalysts revealed that their Au particle sizes (~2.4 nm) remained nearly unchanged before and after propene epoxidation (Figure S15).

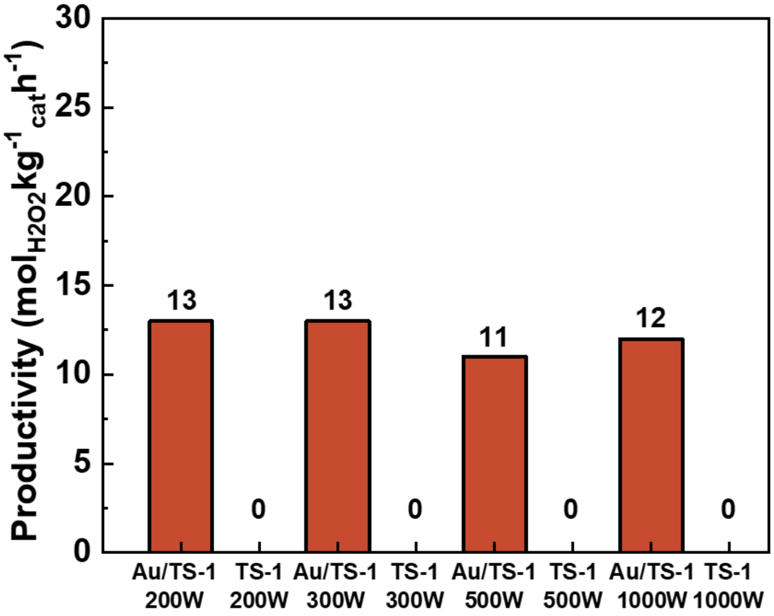

Figure S16 The productivity of H_2O_2 on different Au/TS-1 catalysts. The productivity of H_2O_2 by direct synthesis between H_2 and O_2 on different Au/TS-1 catalysts. H_2O_2 synthesis conditions: Catalyst (0.01g), H_2O (2.9 g), MeOH (5.6 g), 5% H_2/CO_2 (420 psi), 25% O_2/CO_2 (160 psi), reaction time 0.5 h, reaction temperature 2 °C, stirring speed 1200 rpm.

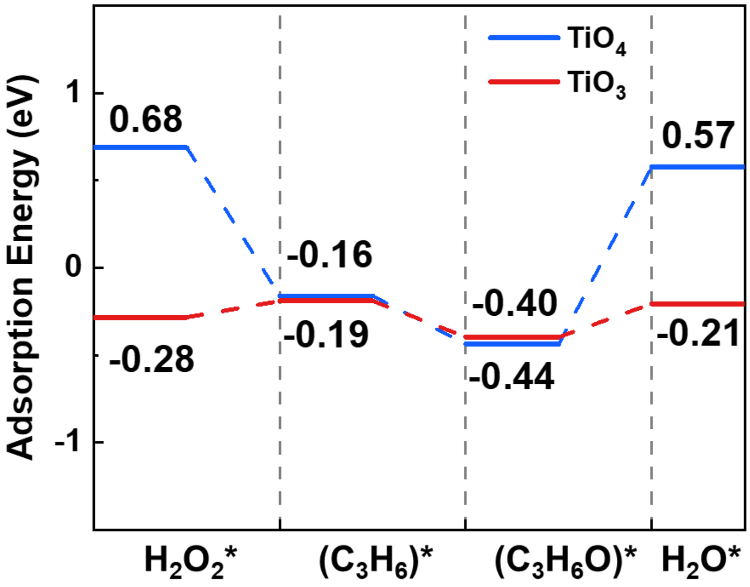

Figure S17 Free energies of adsorption on TiO_4 and TiO_3 sites. Calculated free energies of adsorption for different intermediates on TiO_4 and TiO_3 sites.

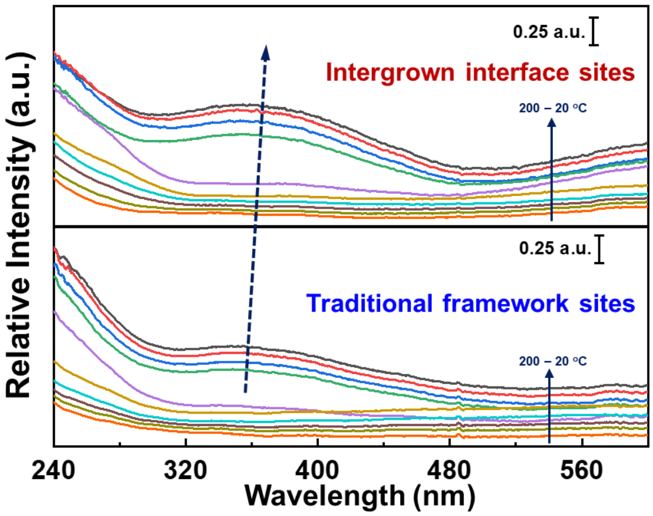

Figure S18 In-situ UV-vis spectra of different catalysts. In-situ UV-vis spectra of Au/TS-1-300W (traditional framework sites) and Au/TS-1-500W (intergrown interface sites) after contact with performed H_2O_2. Experimental conditions: 0.15 g of TS-1 samples were used in conjunction with 0.01 g of H_2O_2, while maintaining a temperature range from 20 to 200°C, with data points collected every 20-degree interval.

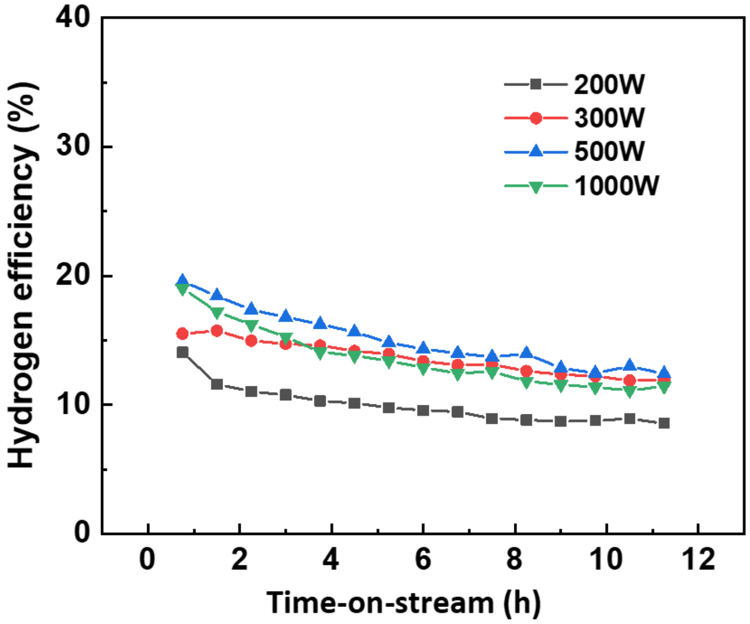

Figure S19 The hydrogen efficiency of different catalysts. The hydrogen efficiency of Au/TS-1-200W, Au/TS-1-300W, Au/TS-1-500W and Au/TS-1-1000W. Direct propene epoxidation with H_2 and O_2 conditions: reaction temperature (200 ^{o}C), catalyst (0.15 g), $H_2/O_2/C_3H_6/N_2$=1:1:1:7, space velocity of 14000 mL•h^{-1}•g_{cat}^{-1}.

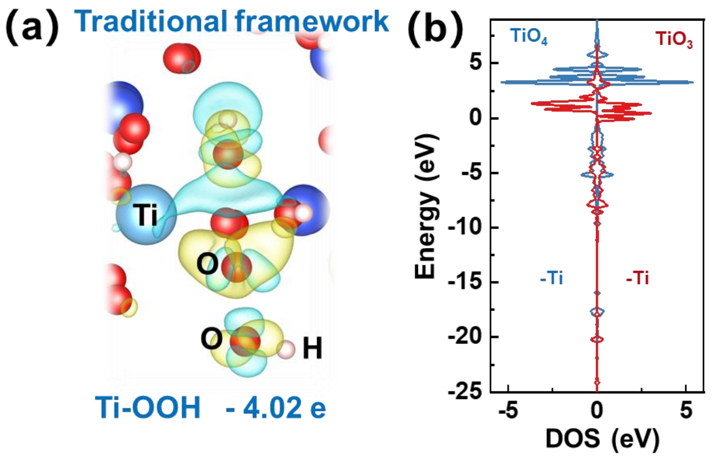

Figure S20 The electronic structure of different Ti sites. (a) The differential charge density plots of H_2O_2 adsorbed on traditional framework sites. (b) The total density of states (DOS) of Ti- atoms on traditional framework and intergrown interface sites.
